# Enhancing the Therapeutic Efficacy of Berberine and Quercetin Through Salt Formulation for Liver Fibrosis Treatment

**DOI:** 10.3390/ijms26052193

**Published:** 2025-02-28

**Authors:** Yangyang Cheng, Haoyang Yu, Sitong Yang, Xiaolian Tian, Mengyu Zhao, Ling Ren, Xiuping Guo, Chujuan Hu, Jiandong Jiang, Lulu Wang

**Affiliations:** Institute of Medicinal Biotechnology, Chinese Academy of Medical Science & Peking Union Medical College, Beijing 100050, China; yy550258560@outlook.com (Y.C.); yuhy1014@163.com (H.Y.); yangst0323@163.com (S.Y.); 18852072531@163.com (X.T.); zhaomengyu85@163.com (M.Z.); renling0225@163.com (L.R.); gxp2000429@163.com (X.G.); hcj20000616@163.com (C.H.)

**Keywords:** liver fibrosis, berberine, quercetin, cocktail therapy

## Abstract

Liver fibrosis, caused by chronic hepatic injury, is a major threat to human health worldwide, as there are no specific drugs available for its treatment. Natural compounds, such as berberine (BBR) and quercetin (QR), have shown the ability to regulate energy metabolism and protect the liver without significant adverse effects. Additionally, combination therapy (the cocktail therapy approach), using multiple drugs, has shown promise in treating complicated conditions, including liver injury. In this study, we prepared a salt formulation of BBR and QR (BQS) to enhance their combined effect on liver fibrosis. The formation of BQS was confirmed using various analytical techniques, including nuclear magnetic resonance spectroscopy (NMR), differential scanning calorimetry (DSC), Fourier-transform infrared spectroscopy (FTIR), powder X-ray diffractometry (PXRD), and scanning electron microscopy (SEM). The results demonstrated that the dissolution efficiency and bioavailability of QR significantly increased in the BQS form, aligning with that of BBR, compared to the physically mixed (BQP) form. Moreover, BQS exhibited a superior inhibitory effect on fibrosis compared to BQP in the human hepatic stellate cell line LX-2 by modulating lipid accumulation, inflammation, apoptosis, and the cell cycle. Furthermore, in a mouse model of hepatic fibrosis induced by methionine and choline-deficient (MCD) diets, BQS demonstrated enhanced anti-fibrotic activities compared to BQP. These findings suggest that BQS holds promise as a potential alternative treatment for liver fibrosis. Importantly, this study provides novel insights into achieving a cocktail effect through the salt formation of two or more drugs. The results highlight the potential of salt formulations in enhancing the therapeutic efficacy and consistent biological processes of drug combinations.

## 1. Introduction

Liver diseases, which result from a multitude of factors, including unhealthy lifestyles, viral infections, genetic susceptibility, and drug-induced injury, pose a significant global health threat. These diseases commonly progress through various stages, starting with the accumulation of lipids, known as nonalcoholic fatty liver disease (NAFLD). Chronic NAFLD can then lead to nonalcoholic steatohepatitis (NASH), cirrhosis, hepatocellular carcinoma (HCC), and ultimately, mortality [1,2]. Liver fibrosis, a reversible progression of cirrhosis, represents a promising target for therapeutic intervention in cirrhosis. However, currently, there is no specific therapy available for liver fibrosis [3]; hence, the search for safe and effective drugs to treat it is of utmost importance.

In recent decades, there has been a growing focus on natural compounds due to their potential for liver protection and low toxicity. Furthermore, the synergistic effects of combining herbs with similar pharmacological properties, often referred to as the cocktail effect, have shown promising outcomes. Historically, cocktail treatments have demonstrated improved efficacy, not only in infectious diseases such as Sudan virus infection [4], human immunodeficiency virus infection [5], Staphylococcus Aureus infection [6] and COVID-19 infection [7], but also in chronic diseases like chronic asthma [8] and Alzheimer’s disease [9]. By reducing side effects and drug resistance, cocktail treatment offers a favorable approach to enhance therapeutic outcomes through the combined effects of multiple drugs.

Berberine (BBR) is an isoquinoline alkaloid extracted from *Coptidis rhizoma*, which is used in traditional Chinese medicine. It possesses diverse biological activities, including anticancer [10], metabolic improvement [11], and antibacterial effects [12]. Recent studies have highlighted the potential of BBR in treating liver fibrosis through various direct and indirect mechanisms. From the direct perspective, BBR can modulate the apoptosis, proliferation, and activation of hepatic stellate cells (HSCs). It induces ferroptosis through ROS-mediated activation of HSCs [13] and promotes HSCs apoptosis by reducing mitochondrial membrane potential [14]. BBR also arrests HSCs in the G1 phase [15] and reduces the expression of matrix metalloproteinases (MMPs) and tissue inhibitors of MMPs (TIMPs), thereby facilitating collagen degradation [16]. Indirectly, BBR improves lipid metabolism [17], reduces endoplasmic reticulum stress [18], modulates gut microbiota [19], inhibits oxidative stress [20] and suppresses inflammation [21], all of which contribute to its anti-fibrotic effect. However, the low intestinal permeability and aqueous solubility of BBR limit its oral bioavailability.

Quercetin (QR), a flavonoid compound widely found in plants and fruits, has limited bioavailability due to its poor solubility, permeability, and stability. Despite these challenges, QR exhibits a wide range of pharmacological actions, including anticancer [22], neuroprotection [23] and liver protection [24]. In the context of liver fibrosis, QR regulates MMP-9 and TIMP-1 to inhibit extracellular matrix formation. It also inhibits autophagy through modulation of the HMGB1-TLRs-NF-kB signaling pathways [25], activates the PI3K/Akt signaling pathway, and suppresses the TGF-β1/Smads signaling pathway [26]. Additionally, QR exhibits liver-protective functions, such as oxidation resistance and anti-inflammatory action, which contribute to its effectiveness in improving liver fibrosis [27].

BBR and QR, derived from natural sources, have demonstrated the potential to improve liver fibrosis through various mechanisms. Moreover, they exhibit a favorable safety profile with minimal adverse effects at standard doses. Interestingly, clinical studies have shown that combining flavonoids with BBR can enhance their pharmaceutical effects [28,29]. A meta-analysis revealed that the combination of BBR and QR improved lipid and glucose levels in patients. Traditional prescriptions incorporating both BBR and QR further support the feasibility and effectiveness of a cocktail therapy approach [30,31,32].

However, the efficacy of BBR and QR is hindered by their disparate dissolution and absorption characteristics. To address this issue and enhance their combined effects, we synthesized a BBR-QR salt (BQS). This formulation aims to improve the bioavailability of both compounds, thereby facilitating their simultaneous absorption. We evaluated its efficacy in treating liver fibrosis through both in vitro and in vivo experiments, anticipating enhanced therapeutic outcomes.

## 2. Results

### 2.1. Synthesis and Characterization of BBR-QR Salt

**Figure 1:**
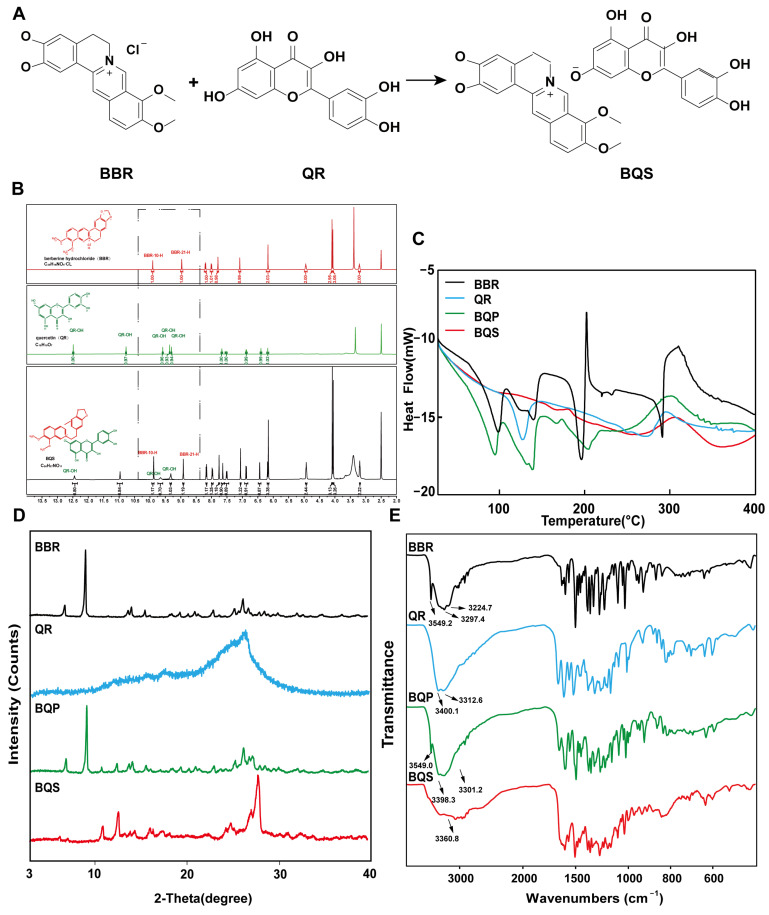
Synthesis and characterization of BQS (Berberine-quercetin salt). (**A**) The synthetic route of BQS. (**B**) The ^1^H NMR spectra of the BBR (Berberine), QR (Quercetin) and BQS. (**C**) The DSC spectra of BBR, QR, BQP (Berberine-quercetin physically mixed), and BQS. (**D**) The PXRD of BBR, QR, BQP, and BQS. (**E**) The FTIR of BBR, QR, BQP, and BQS.

As shown in Figure 1A, BQS was successfully synthesized using BBR and QR. The BBR acts as the cation, and the QR as the anion, forming the BQS salt through acid-base interactions. The obtained BQS was 7.4 g, and the yield was 87%. The ^1^H NMR, FTIR, PXRD, DSC and SEM were used to prove the formation of BQS.

#### 2.1.1. Hydrogen-1 Nuclear Magnetic Resonance (^1^H NMR) Results

As seen in Figure 1B, the synthesized compound BQS was characterized as a mono-substituted product of BBR and QR based on ^1^H NMR analysis. The molecular formula of BQS is C_35_H_27_NO_14_, and its ^1^H NMR spectrum indicates the presence of 27 hydrogen atoms, consistent with this structure. When comparing the ^1^H NMR spectra of BQS, BBR, and QR, it is observed that the chemical shifts in BQS include signals corresponding to both BBR and QR, indicating the combination of these two compounds. Importantly, the ^1^H NMR spectrum of BQS lacks one specific proton signal near δ 9.33, which corresponds to one of the hydroxyl groups of QR. This absence is indicative of the formation of salt between the protonated nitrogen of BBR and a single hydroxyl group of QR, which conclusively supports the formation of a 1:1 mono-substituted quercetin-berberine salt (Supplementary Figure S1).

#### 2.1.2. Differential Scanning Calorimetry (DSC) Results

We can conclude, from the DSC results shown in Figure 1C, that the curve of BQS is different from BQP, BBR, and QR, respectively. To be specific, BBR had three peaks at different temperatures: 98.37 °C (free water), 139.48 °C (crystal water), and 196.20 °C (the melting point peak). There was an endothermic peak at 278.42 °C in the QR curve, which was similar to its melting point. As for the curve of BQP, it appeared to be a superposition of two respective curves. Three exothermic peaks were observed at 94.91 °C, 139.09 °C, and 150.08 °C. However, there were no characteristic peaks in the curve of BQS, which was another piece of evidence for the formation of the amorphous substance.

#### 2.1.3. Powder X-Ray Diffraction (PXRD) Results

Figure 1D represents the PXRD results. The diffractogram of BBR and QR showed sharp peaks of crystals. The spectrum of BQP consisted of the superposition of BBR and QR. The diffraction spectra of BQS had no sharp peaks, displaying a typical diffuse diffraction halo of the crystalline state, indicating the formation of an amorphous substance, which may have contributed to the improvement of the solubility and bioavailability.

#### 2.1.4. Fourier-Transform Infrared Spectroscopy (FTIR) Evaluation

Figure 1E represents the FTIR spectra of BBR, QR, BQP, and BQS. As illustrated in the picture, the infrared spectrum of BQS had no characteristic absorption peaks of BBR and QR at 3200–3600 cm^−1^, indicating that the O-H stretching vibration disappeared. The spectrum of BQP had the infrared signatures of the two pure drugs. This difference between the spectra of BQS and BQP supports the hypothesis that BQS is a novel compound resulting from the synthesis of the two drugs. A deviation of ±2 cm^−1^ was allowed in the infrared spectra.

#### 2.1.5. Scanning Electron Microscopy (SEM) Results

**Figure 2:**
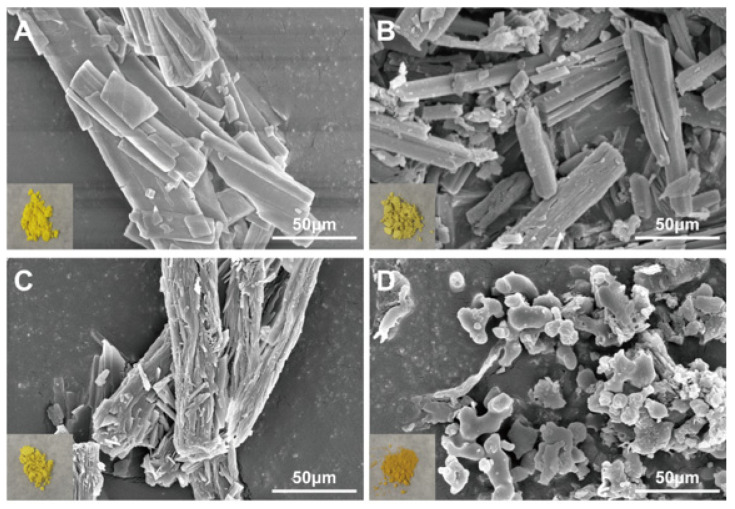
SEM image and appearance of BBR, QR, BQP, and BQS. (**A**) BBR, (**B**) QR, (**C**) BQP, (**D**) BQS.

The appearance of BQS is deep yellow, which is obviously different from the appearance of BQP, BBR, and QR (Figure 2). As shown in Figure 2, both BBR and QR are presented as elongated rod-shaped crystals. BQP is also presented as elongated rod-shaped crystals. In comparison, BQS was distinctive in form, showing no features identical to BBR and QR at all.

To sum up, these characterization means have proved that we synthesized BQS successfully.

### 2.2. BQS Improved the Dissolution and Bioavailability of QR

**Figure 3:**
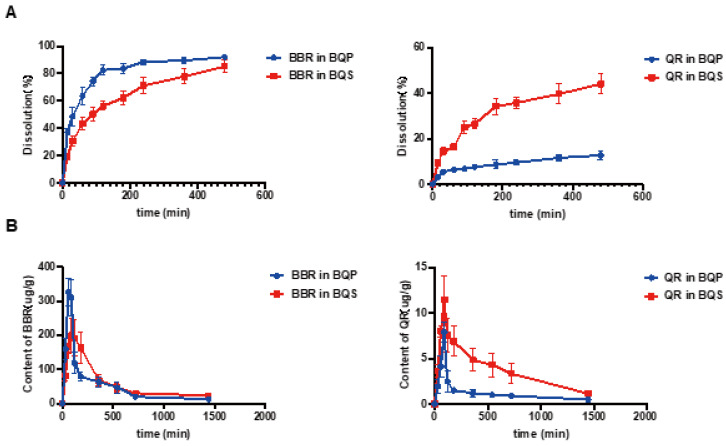
The dissolution and bioavailability of BBR and QR. (**A**) The dissolution of BBR and the dissolution of QR. (**B**) BBR content in the liver, QR content in the liver, Mean ± SEM, n = 5.

The dissolution studies showed that (Figure 3A) BBR in BQP dissolved well with rapid release within 2 h, while a sustained release of BBR was found in BQS. The dissolution of QR was markedly promoted after the formation of salt by combining it with BBR. It was noteworthy that the salt formation realized synchronous release of BBR and QR.

LC-MS/MS was used to determine the concentration of BBR and QR in the livers of mice who were given the drugs via intragastric administration. Figure 3B represents the concentrations of BBR and QR, respectively. As shown in the Figure 3B, the concentration of BBR remains higher in the BQS group than in the BQP group, which may lead to a long-lasting therapeutic effect. The area under the curve for the BQS group was 17% higher than that for the BQP group. Furthermore, the concentration of QR was markedly promoted after salt formation with BBR (approximately 2.2-fold higher than that observed in the BQP group).

The dissolution and pharmacokinetic properties of QR were improved by salt formation.

### 2.3. BQS Ameliorated Liver Fibrosis In Vitro

The activation of HSCs plays a vital role in liver fibrosis. After liver injury, HSCs were activated and produced extracellular matrix (ECM) and pro-inflammatory mediators, triggering progressive liver fibrosis [33]. Both BBR and QR can suppress inflammation [34] in parenchymal cells. Furthermore, numerous studies have proven that BBR is able to induce apoptosis and inhibit proliferation in HSCs [14,35]. It is possible to combine BBR and QR for a better therapeutic effect [28,29].

#### 2.3.1. BQS Inhibited Lipid Accumulation and Inflammation

In the process of liver fibrosis, the undue accumulation of lipids and inflammation are two risk factors. Thus, we established a HepG2 hepatic steatosis model by treating the cells with 0.5 mmol/L sodium oleate to investigate the effects of BQS on lipid accumulation and inflammation.

**Figure 4:**
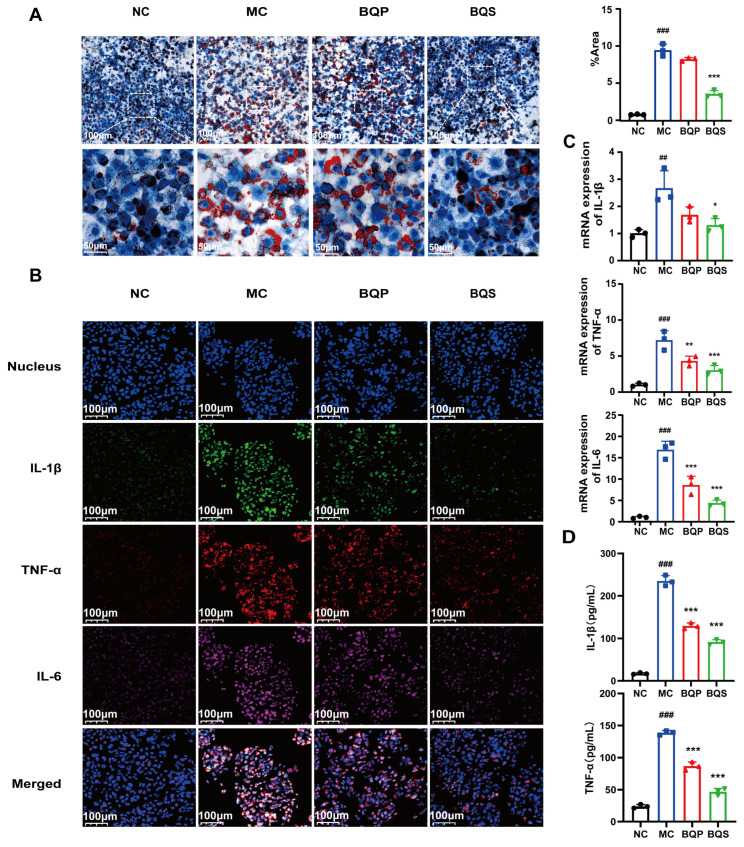
Lipid accumulation and inflammatory expression in HepG2 cells. (**A**) The results of Oil Red O staining. And the quantification of Oil Red O staining. Mean ± SEM, n = 3. (**B**) Immunofluorescence analysis showing DAPI (blue), IL-1β (green), TNF-α (red), and IL-6 (pink) in HepG2 cells of each group. (**C**) The relative mRNA level of IL-1β, TNF-α, IL-6. (**D**) The productions of IL-1β and TNF-α in supernatants of LX-2 cells were measured by ELISA. Mean ± standard error of the mean (SEM), n = 3. *: vs. MC group; ^#^: vs. NC group. Significant differences are indicated as * *p* < 0.05, ** *p* < 0.01, *** *p* < 0.001; ^##^ *p* < 0.01, ^###^ *p* < 0.001.

As shown in Figure 4A, there are a few lipid droplets in the NC group; however, the lipid droplets in the MC (model control) group are much more frequent than those of the NC group. This phenomenon was attenuated not by BQP (10 μg/mL of BBR and 9.0 μg/mL of QR) but by BQS (19 μg/mL). Excessive lipid accumulation may trigger the development of NAFLD, which may eventually progress to liver fibrosis [36]. Thus, inhibiting lipid accumulation is a promising method for the prevention and treatment of liver fibrosis. BQS exhibited a remarkable effect in this area.

Inflammation plays an imperative role in the progress of liver fibrosis [37]. Modulating inflammation is a promising method for the treatment of liver fibrosis. TNF-α, IL-1β, and IL-6 are common inflammation factors, and they were therefore used to study the anti-inflammatory effects of the drugs (Figure 4B). TNF-α was dyed red, IL-1β was dyed green, and IL-6 was dyed pink. Compared to the NC group, the intensity of fluorescence of the MC group was much higher, indicating the success of our model. BQP attenuated the inflammation to some extent. BQS improved inflammation by a large margin to almost the same level as the NC group. The qPCR and ELISA results further confirmed the consistent trend observed in the treatment outcomes (Figure 4C,D).

Given the favorable effects of BQS on inhibiting lipid accumulation and inflammation, it may become a curative strategy for liver fibrosis.

#### 2.3.2. BQS Alleviated Extra Collagen Formation

**Figure 5:**
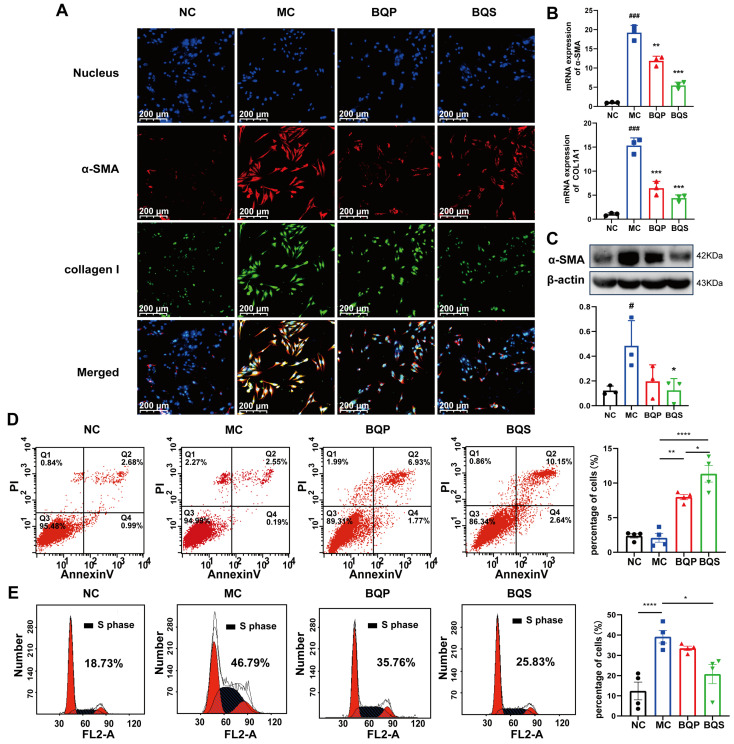
The in vitro effects of BQS on collagen formation and the cell cycle evaluated in LX-2 cells. (**A**) Immunofluorescence analysis showing DAPI (blue), α-SMA (red), collagen I (green) in LX-2 cells of each group.(**B**) The relative mRNA level of α-SMA, COL1A1 in LX-2 cell, Mean ± standard error of the mean (SEM), n = 3. (**C**) Protein levels of α-SMA were detected in LX-2 cells by western blot. (**D**) The results of apoptosis of LX-2 cells, Mean ± standard error of the mean (SEM), n = 4. (**E**) Cell cycle of LX-2 cells. Mean ± standard error of the mean (SEM), n = 4. *: vs. MC group; ^#^: vs. NC group. Significant differences are indicated as * *p* < 0.05, ** *p* < 0.01, *** *p* < 0.001; **** *p* < 0.0001; ^#^ *p* < 0.05, ^###^ *p* < 0.001.

As downstream proteins of the TGF-β signaling pathway [38,39], α-SMA and collagen I are markers of the occurrence of fibrosis. In this study, we measured the expression of α-SMA and collagen I in LX-2 cells (Figure 5A). α-SMA was dyed red, and collagen I was dyed green. As the picture shows, little α-SMA and collagen I were expressed in the NC group. While inducing the TGF-β pathway, the expression of α-SMA and collagen I evidently increased in the MC group, indicating that our model was successful. When we came to BQP and BQS, the intensity of fluorescence in BQP and BQS was lower than that in the MC group. The difference was that the intensity of fluorescence in BQP only attenuated a little, while the intensity of fluorescence in BQS reduced a lot. This phenomenon enlightens us that BQS had a much better anti-fibrosis effect than BQP. The PCR and Western blot analyses showed similar results (Figure 5B,C).

#### 2.3.3. BQS Modulated Proliferation and Apoptosis

As an important cell in the development of liver fibrosis, activated LX-2 is an important target for treatment. Managing the number of activated HSCs is an ideal treatment strategy. In this experiment, we investigated the pro-apoptotic and anti-proliferative effects of BQS.

Previous studies have shown that inducing the apoptosis of activated HSCs is beneficial to the amelioration of liver fibrosis [40,41]. Flow cytometry was used to explore the apoptosis and cell cycle of the LX-2 cells incubating with BQP and BQS. The percentage of apoptotic cells in each group is 2.37 ± 0.61, 2.09 ± 1.27, 7.98 ± 0.76 and 11.34 ± 2.4 (Figure 5C). The pro-apoptotic effect of BQS was obviously stronger than BQP, which may lead to better therapeutic effects for liver fibrosis.

Moreover, besides promoting apoptosis, the BQS was also capable of inhibiting the proliferation of LX-2 cells. Cells in the synthesis (S) phase of cell division have the strongest division potential. The more cells in this phase, the more likely they are to divide. As displayed in Figure 5D, the cells in the S phase accounted for 18.73%, 46.79%, 35.76%, and 25.83% of the NC group, MC group, BQP group, and BQS group, respectively. The BQP demonstrated a small inhibition effect on the proliferation of LX-2 cells, while BQS showed a significant effect. Overall, BQS was capable of inducing the apoptosis of LX-2 cells and inhibiting their proliferation. BQP, however, did not have these capabilities. In the process of liver fibrosis, HSCs are activated by TGF-β secreted by Kuffer cells, and then they secrete large amounts of TGF-β themselves and transform into myofibroblasts to secrete collagen and other proteins to form the ECM [42]. By inducing the apoptosis of HSCs and inhibiting their proliferation, the progression of liver fibrosis can be alleviated. Thus, we speculate that our BQS exhibits an excellent anti-fibrosis effect in vivo, which lays the foundation for our following research.

### 2.4. BQS Improved Liver Fibrosis In Vivo

**Figure 6:**
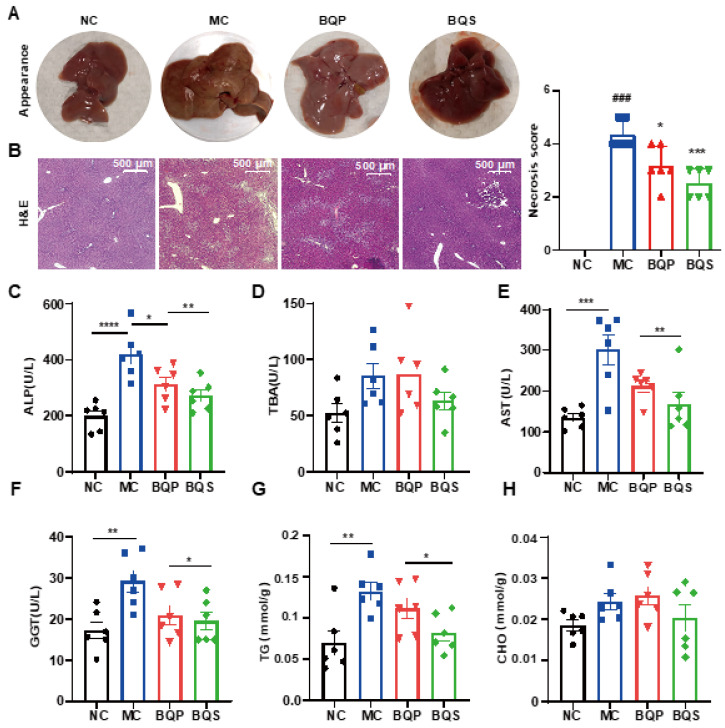
The hepatoprotective effects of BQS in mice model of liver fibrosis. (**A**) The appearances of mice liver tissue in different groups. (**B**) H and E staining of mouse liver in each group. (**C**–**F**) Blood biochemical results (ALP, TBA, AST and GGT). (**G**,**H**) The TG and CHO content in mice liver. Mean ± standard error of the mean (SEM), n = 6, *: vs. MC group; ^#^: vs. NC group. * *p* < 0.05; ** *p* < 0.01; *** *p* < 0.001; **** *p* < 0.0001. ^###^ *p* < 0.001.

Encouraged by the results of in vitro studies, the potential therapeutic effect of BQS on liver fibrosis was next investigated in a mice model. Methionine and Choline Deficit (MCD) diets are recognized methods for modeling liver fibrosis. We took photographs of livers from typical samples from four groups of animals (Figure 6A). The appearance of the BQP group was somewhere between the NC and MC groups. The appearance of the BQS group was similar to the NC group, indicating a better effect of BQS than BQP, which was also proven by other results displayed in the following sections.

#### 2.4.1. BQS Protected Liver Function

H and E staining (Figure 6B) showed that the histological structure of the liver was severely damaged in the MC group, significantly improved in the BQS group, and slightly improved in the BQP group. The indicators ALP and TBA were improved for the BQS group only (Figure 6C,D), meaning that BQS alleviated liver damage. Moreover, BQS also improved the levels of ALT, AST, and GGT, which means that BQS can contribute to liver function, indicating that BQS has an improvement effect on liver fibrosis, while BQP showed influence only on AST and GGT (Figure 6E,F). Lipid accumulation is another risk factor for liver fibrosis [43]. We compared the lipid accumulation in the liver of different groups (Figure 6G,H). The results showed that BQP and BQS reduced the TG levels, while the CHO level was only reduced by BQS. These changes remind us that BQS can inhibit lipid accumulation, which is beneficial for anti-inflammation and anti-fibrosis purposes.

#### 2.4.2. BQS Ameliorated Liver Fibrosis

**Figure 7:**
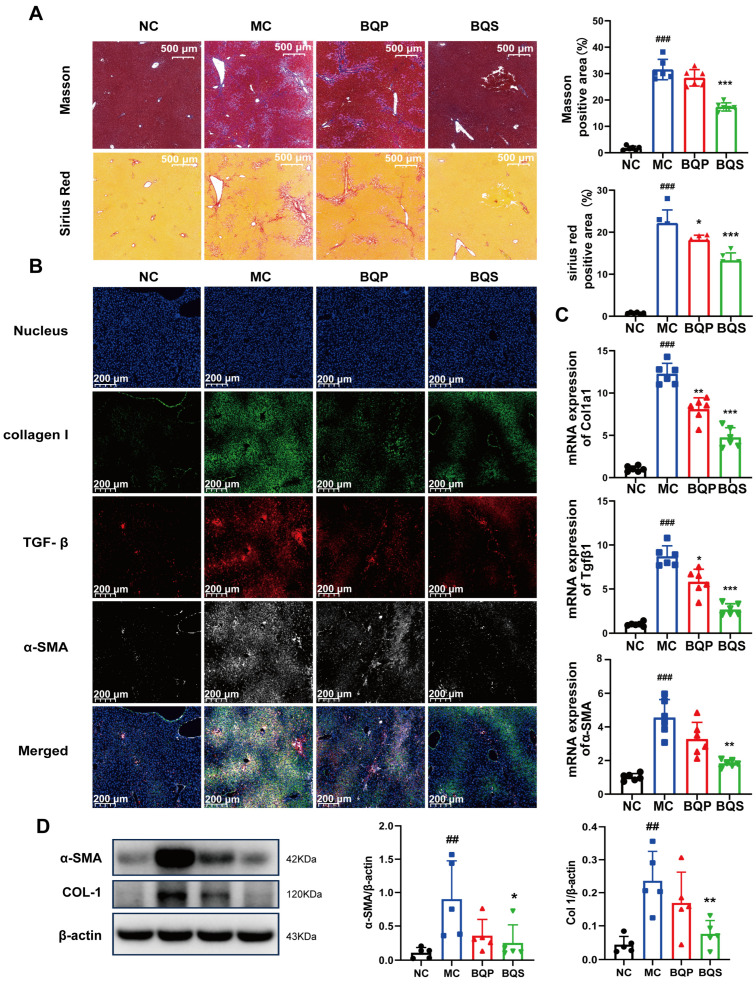
The antifibrotic effects of BQS in mice model of liver fibrosis. (**A**) Masson and Sirius Red staining of mice liver in each group. (**B**) Immunofluorescence analysis showing DAPI (blue), collagen I (green), TGF-β (red), and α-SMA (gray) of mouse liver in each group.(**C**) The relative mRNA level of Col1a1, Tgfβ, α-SMA in mice liver. (**D**) Protein levels of α-SMA COL-1 were detected in mice liver by western blot. Mean ± standard error of the mean (SEM), n = 5 or n = 6, *: vs. MC group; ^#^: vs. NC group. * *p* < 0.05, ** *p* < 0.01; *** *p* < 0.001; ^##^ *p* < 0.01, ^###^ *p* < 0.001.

The result of Masson staining is shown in Figure 7A. After staining, the collagenous fiber became blue, enabling its visualization. Sirius Red staining was also used to visualize collagenous fiber and evaluate the degree of fibrosis. It is obvious that BQS reduced the accumulation of collagen in the liver; however, BQP showed nearly no effect. Meanwhile, we can draw the same conclusion from Figure 7B, where the TGF-β was dyed red, collagen I was dyed green, and α-SMA was dyed gray. These three markers, which are closely related to fibrosis, were significantly inhibited by BQS. The PCR analysis showed a similar result (Figure 7C). Western blot further confirmed these findings, and BQS exhibits a more potent anti-fibrotic effect (Figure 7D). These results are highly similar to the in vitro results.

#### 2.4.3. BQS Alleviated Inflammation in Liver Tissue

**Figure 8:**
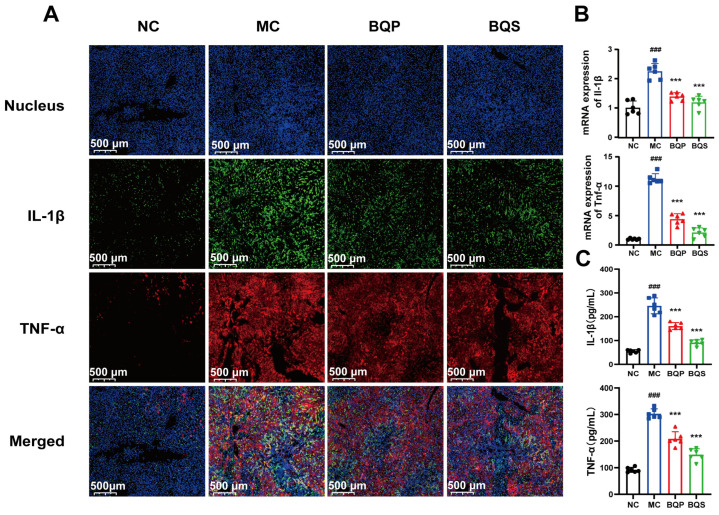
The image of two inflammatory factors of animals. (**A**) Immunofluorescence analysis showing DAPI (blue), IL-1β (green), TNF-α (red) of mouse liver in each group. (**B**) The relative mRNA level of the inflammatory marker IL-1β, TNF-α in mice liver tissue. (**C**) The productions of IL-1β and TNF-α in supernatants of indicated cells were measured by ELISA. Mean ± standard error of the mean (SEM), n = 6. *: vs. MC group; ^#^: vs. NC group. Significant differences are indicated as *** *p* < 0.001; ^###^ *p* < 0.001.

The results of the immunohistochemistry analysis (Figure 8A) showed that inflammation was suppressed by BQS. TNF-α was dyed red, and IL-1β was dyed green. After modeling, there were significant differences between the MC group and the NC group. Both BQP and BQS improved the inflammation, but BQS had a more dramatic effect. The PCR and ELISA analyses showed similar results (Figure 8B,C).

Taken together, the in vivo experiments showed the same results as the in vitro experiments, implying that our BQS could exert anti-fibrotic pharmacological effects in vivo.

In every aspect we investigated, BQS had better effects than BQP.

### 2.5. Network Pharmacology Analysis and Mechanism Validation

**Figure 9:**
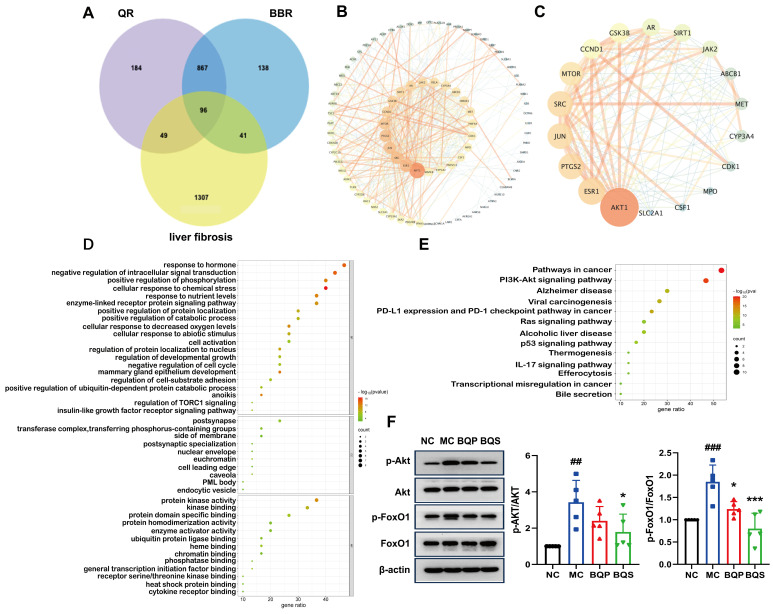
Network pharmacological analysis and protein pathway validation of BQS in fibrosis. (**A**) Acquisition of drug–disease intersection targets. (**B**) Construction of protein interaction network (PPI). (**C**) Core target screening and visualization analysis. (**D**) Enrichment analysis of gene ontology (GO). (**E**) Enrichment analysis of Kyoto Encyclopedia of Genes and Genomes (KEGG). (**F**) The protein expression of p-Akt/Akt and p-FoxO1/FoxO1 in mouse liver tissue was evaluated by Western blotting, with β-actin used as the control. Mean ± SEM, n = 5. *: vs. MC group; ^#^: vs. NC group. Significant differences are indicated as * *p* < 0.05, *** *p* < 0.001; ^##^ *p* < 0.01, ^###^ *p* < 0.001.

In the current investigation, a network pharmacological approach was employed to delineate the molecular mechanisms underpinning the therapeutic effects of BQS on liver fibrosis. A comprehensive intersectional target analysis identified a set of 96 potential targets associated with both BQS components and liver fibrosis (Figure 9A). The subsequent construction of a protein–protein interaction (PPI) network using the STRING database, and analysis with Cytoscape 3.10.1, identified 87 nodes and 472 edges, representing a complex interaction landscape (Figure 9B). Core targets were extracted using Centiscape 2.2, with visual analytics further refining these to 18 pivotal nodes within the network (Figure 9C). Gene ontology (GO) functional annotations indicated the significant modulation of molecular functions following BQS treatment, particularly noting enhancements in protein kinase activity, receptor binding, and oxidoreductase activity, which are crucial for cellular signaling and metabolic regulation (Figure 9D, top panel). Moreover, the Kyoto Encyclopedia of Genes and Genomes (KEGG) pathway analysis highlighted the importance of the ’Pathways in Cancer’ and ’PI3K-Akt signaling pathway’ in the mechanistic action of BQS, suggesting its central role in the amelioration of hepatic fibrosis (Figure 9E).

Protein expression levels of phospho-Akt (p-Akt) and phospho-FoxO1 (p-FoxO1) in hepatic tissues were quantified using Western blotting assays. As shown in Figure 9F, comparative analysis revealed that in contrast to the NC group, the phosphorylation of both Akt and FoxO1 was significantly elevated in the MC group, indicating enhanced activity within this signaling axis. Notably, treatment with BQS markedly attenuated these phosphorylation levels. These findings suggest that the therapeutic effect of BQS on liver fibrosis can be attributed to the inhibition of the Akt/FoxO1 signaling pathway. Furthermore, BQS demonstrated superior efficacy in modulating these pathways compared to BQP.

## 3. Discussion

Liver fibrosis is a progressive medical condition characterized by the excessive accumulation of ECM proteins, notably collagen [44]. This pathological buildup disrupts liver architecture and impairs its function. The condition commonly arises from chronic liver damage due to factors such as hepatitis infections, alcohol abuse, NAFLD, and other metabolic disorders. As fibrosis advances, it may progress to cirrhosis—a severe form of liver scarring that significantly impairs liver function and escalates the risk of liver failure and liver cancer. Globally, the incidence of liver fibrosis is increasing, primarily due to escalating rates of obesity, diabetes, and metabolic syndrome, which are key contributors to NAFLD, now recognized as one of the most prevalent causes of liver disease worldwide [45]. Consequently, the rising prevalence and severity of liver fibrosis underscore the urgent need for the development of more effective treatments.

BBR, an isoquinoline alkaloid, has been increasingly recognized for its hepatoprotective effects, including its efficacy against liver fibrosis. Research has shown that BBR ameliorates liver fibrosis through multiple mechanisms. It possesses anti-inflammatory properties and counteracts oxidative stress, which are crucial steps in halting the progression of the disease. Additionally, BBR inhibits the activation of HSCs, which is central to the deposition of fibrotic tissue [14,46]. Furthermore, it regulates metabolic pathways, notably by improving insulin resistance and modulating lipid metabolism, thus offering a protective effect on the liver [19]. This multifaceted strategy highlights the potential of BBR as a therapeutic agent in the management of liver fibrosis.

QR has been demonstrated to possess potent anti-inflammatory properties. Studies suggest that in liver fibrosis, QR significantly reduces the expression of pro-inflammatory cytokines, including TNF-α and IL-6. Moreover, it inhibits the activation of HSCs and promotes their apoptosis. QR exerts its therapeutic effects by modulating several critical signaling pathways associated with liver fibrosis, notably the TGF-β pathway [47]. Such modulation results in the downregulation of α-SMA and collagen expression, effectively impeding the progression of liver fibrosis. Recognizing the established hepatoprotective effects of BBR and QR, which operate via distinct mechanisms and are inspired by the principles of principal-assistant synergy and cocktail therapy from traditional Chinese medicine, we proposed a combination of these two compounds to enhance therapeutic outcomes. This strategic combination aims to harness the complementary mechanisms of BBR and QR, potentially offering a more effective therapeutic profile for liver protection.

The formation of salts from two different compounds, termed pharmaceutical salt preparation, offers numerous advantages, particularly within the pharmaceutical sector. This technique is widely used to enhance the physicochemical properties and biological efficacy of drugs. In this study, we initially focused on the synthesis and characterization of BQS. The synthesis yielded 7.4 g of BQS at an efficiency of 87%, as confirmed by various analytical techniques, including ^1^H NMR, FTIR, PXRD, DSC, and SEM. The ^1^H NMR data confirmed the successful synthesis, evidenced by a reduction in the number of hydrogen peaks from 28 in BQP to 27 in BQS, with peak shifts and shape alterations indicating interactions between BBR and QR in the salt form. DSC analysis revealed distinct thermal behavior of BQS, differing from BQP and individual components by showing no characteristic peaks, indicative of the formation of an amorphous substance. This finding was corroborated by PXRD results, which displayed diffuse diffraction patterns typical of amorphous materials, likely enhancing solubility and bioavailability. The FTIR spectra further validated the synthesis, with the absence of characteristic O-H stretch vibrations in BQS, suggesting interactions that alter typical absorption peaks of the individual components. The distinct deep yellow color of BQS, different from either component or their physical mixture, visually confirms these findings.

QR is renowned for its potent antioxidant and anti-inflammatory properties. It may reduce inflammatory responses, thereby minimizing damage to the intestinal barrier and enhancing the absorption of BQS. BBR, known for its synergistic pharmacological effects with QR, is a substrate for various efflux transporters, such as P-glycoprotein (P-gp), which expels BBR from cells, reducing its absorption and bioavailability. QR has been shown to inhibit the activity of these transporters, thereby increasing the retention and intestinal absorption of BBR [48,49]. Additionally, QR is known to inhibit certain enzymes in the CYP450 family, particularly CYP3A4 and CYP2D6, which are essential for metabolizing many drugs, and BBR is also a substrate for these enzymes [50]. By inhibiting these enzymes, QR can slow the metabolism of BBR, resulting in elevated plasma concentrations. LC-MS/MS analysis has demonstrated that BQS achieves higher and more sustained concentrations in liver tissue, suggesting improved pharmacokinetic properties compared to BQP. This improvement is reflected in both accelerated peak times and higher AUC values for BQS, indicating a more prolonged therapeutic effect. Overall, the successful synthesis and enhanced characteristics of BQS underscore its potential as a more effective therapeutic formulation, combining the benefits of BBR and QR through advanced pharmaceutical techniques.

In the in vitro experiment assessing the anti-hepatic fibrosis effects of BQS, pharmacological evaluations demonstrated that BQS modulates lipid accumulation, inflammatory responses, collagen formation, and cell proliferation. Experimental results showed a significant reduction in lipid droplets and the expression of inflammatory cytokines (TNF-α, IL-1β, and IL-6) in HepG2 cells, as confirmed by fluorescence staining and qPCR analysis. These findings are particularly significant considering the pivotal roles of lipid accumulation and inflammation in the progression of NAFLD and fibrosis. Additionally, BQS was effective in reducing collagen and α-SMA expression in LX-2 cells, thereby attenuating ECM formation, which is crucial in fibrogenesis. BQS not only demonstrated superior anti-inflammatory effects compared to BQP but also significantly inhibited LX-2 cell proliferation and induced apoptosis. Cell cycle analysis indicated a notable reduction in the proportion of cells in the S phase after BQS treatment, underscoring its potent effect on inhibiting cell division. Furthermore, BQS significantly increased the proportion of apoptotic cells compared to either individual compounds or BQP. By targeting multiple biological pathways and modulating the activation of HSCs and fibrosis, BQS shows promise as a potent anti-fibrotic compound. Therefore, further in vivo studies are warranted to validate its efficacy and elucidate its mechanism of action in the treatment of liver fibrosis.

In vivo experiments utilizing a MCD diets-induced mouse model of liver fibrosis demonstrated that BQS exhibits potent therapeutic effects. Results, including liver photographs and histological stains (Masson and Sirius Red), indicated that BQS significantly reduced collagen accumulation and improved the histological architecture of the liver compared to the BQP group and the MC group. Furthermore, BQS notably decreased key fibrotic markers, such as TGF-β, collagen I, and α-SMA, underscoring its robust anti-fibrotic properties. Additional analyses confirmed that BQS preserved liver function, evidenced by improved biochemical markers, including ALP, TBA, ALT, AST, and GGT. Remarkably, BQS outperformed BQP, which exhibited limited efficacy. Moreover, BQS effectively inhibited lipid accumulation—a critical risk factor for liver fibrosis—and demonstrated superior efficacy in reducing TG and CHO levels in liver tissue compared to BQP. The immunohistochemical findings and qPCR analysis further validated that BQS significantly reduced inflammation in liver tissues, evidenced by the marked suppression of inflammatory cytokines such as TNF-α and IL-1β. The in vivo results closely mirrored those observed in vitro, suggesting consistent anti-fibrotic effects of BQS across both experimental settings.

The compounding of Chinese medicines follows the complex traditional principles of ’four qi and five flavors’ alongside the roles of ’ruler, minister, auxiliary, and envoy.’ These principles guide the alteration of medicinal properties and toxicity in herbal combinations. Considering the crucial role of herb pairing in formulations, this article introduces a novel method that refines herb preparation and decoction to potentially enhance the efficacy of the resulting compounds. Specifically, we propose a salt form of BBR and QR, termed BBR–QR salt. A bioinformatics approach was employed to identify disease-related targets and pathways to substantiate the rationality of this drug pairing. Liver fibrosis, characterized by chronic inflammatory damage, currently lacks specific pharmacological treatments, with general strategies focusing on addressing the underlying cause. Previous studies have indicated that reducing lipid droplet aggregation, alleviating inflammation, and promoting apoptosis could positively affect the progression of liver fibrosis. Our bioinformatics analysis underscores the therapeutic potential of BQS, identifying 96 potential targets associated with the components of BQS and liver fibrosis. Subsequent PPI network construction revealed a complex interaction landscape with 18 pivotal nodes. Functional annotations and pathway analysis, especially targeting the ’Pathways in cancer’ and ’PI3K-Akt signaling pathway’, suggest that BQS primarily mitigates hepatic fibrosis through these pathways.

The Akt/FoxO1 signaling pathway critically influences liver fibrosis by modulating cell survival and proliferation [51]. Phosphorylation of FoxO1 by Akt promotes fibrogenesis, allowing fibrogenic cells, such as HSCs, to survive and proliferate, thereby contributing to the accumulation of scar tissue. This progression can lead to severe hepatic conditions, including cirrhosis and liver failure. Under normal conditions, FoxO1 regulates the cell cycle and inflammation; however, its inactivation through phosphorylation triggers uncontrolled cell growth and heightened inflammation, further exacerbating liver damage [52]. Moreover, FoxO1 is involved in metabolic regulation, and its dysfunction is linked to metabolic disorders associated with liver diseases, such as steatosis and NAFLD, thereby increasing the risk of fibrosis [53,54]. Therapeutically targeting this pathway with inhibitors that block FoxO1 phosphorylation or restore its function may effectively reduce liver fibrosis by diminishing HSC activation, enhancing apoptosis, and alleviating inflammation. Such an approach holds potential for the development of new treatments for liver fibrosis. Western blot analysis has confirmed that BQS modulates the Akt/FoxO1 signaling pathway, demonstrating enhanced efficacy in regulating this pathway compared to BQP.

## 4. Materials and Methods

### 4.1. Materials

Berberine hydrochloride (BBR, MW: 371.81) was purchased from Nanjing Zelang Biological Technology Co., Ltd. (Nanjing, China); quercetin (QR, MW: 302.24) was purchased from Aladdin (Shanghai, China); the HepG2 cell line was obtained from the Cell Resource Center, Peking Union Medical College (Beijing, China). The human HSC line LX-2 was obtained from the Shanghai Institutes for Biological Sciences (Shanghai, China). Trypsin EDTA (0.25%), cell culture media, penicillin/streptomycin, and FBS were obtained from Thermo Fisher Scientific (Waltham, MA, USA). All other reagents were of analytical grade.

Male 6-week-old specific-pathogen-free (SPF) C57BL/6N mice were obtained from Beijing Vital River Laboratory Animal Technology Co., Ltd. (Beijing, China). All experimental procedures were approved by the ethics committee of the Institute of Medicinal Biotechnology, Chinese Academy of Medical Sciences and Peking Union Medical College (Beijing, China; No. IMB-20231016D1).

### 4.2. Preparation and Characterization of BBR–QR Samples

First, we dissolved 5 g BBR hydrochloride in 300 mL of water at a temperature of 80 °C until we obtained a clear and transparent light yellow solution. Second, 4.06 g QR was added to a mixed solution of water and ethanol (150 mL: 450 mL); 15 mL of sodium hydroxide aqueous solution containing 0.54 g of sodium hydroxide was also added to this system. Finally, the BBR solution was added, drop by drop, to the system prepared in the previous step while stirring at room temperature. This resulted in the formation of deep yellow precipitation. We continued stirring the mixture at room temperature for one hour after the dropwise addition was complete. Next, we let the sample stand for two hours, then filtered it to obtain the deep yellow precipitation. We left the precipitation at room temperature for two days and then dried it at 50 °C for five hours. This yielded the BBR–QR sample.

Additionally, a physical mixture of BBR and QR was prepared by grinding 9.2 g of BBR hydrochloride with 7.5 g of QR in a mortar until they were thoroughly mixed. The resulting solid, weighing 16.4 g, represented the physical mixture of BBR and QR.

#### 4.2.1. NMR Analysis

A Bruker ASCEND 300 spectrometer was used to record the ^1^HNMR of BBR, QR, BQP, and BQS. The instrument was set to standard mode, 500 MHz, and the sample was dissolved in DMSO.

#### 4.2.2. DSC Analysis

A differential scanning calorimeter (DSC1, Mettler Toledo Zurich, Switzerland) was used to measure the thermodynamic characteristics of the obtained samples. The samples were accurately weighted. The instrument was heated from 30 °C to 800 °C at 10 °C/min in a nitrogen atmosphere of 50 mL/min.

#### 4.2.3. FTIR Analysis

We accurately weighed 10 mg each of BBR, QR, BQP, and BQS for Fourier-transform infrared spectra (Nicolet 5700, Waltham, MA, USA) measurement in the spectral range of 4000–400 cm^−1^. All samples were mixed with KBr at a 1/100 ratio (*w*/*w*) and formed in the KBr disks.

#### 4.2.4. PXRD Analysis

We accurately weighed BBR, QR, BQP, and BQS, which were placed on a vitreous sample holder in a D8 advance X-ray diffractometer with Cu/Ka radiation (λ 0.154 nm) operating at 40 kV, 30 mA while monitoring the reflection angle 2θ from 3° to 40° at a scan speed of 10°/min.

#### 4.2.5. SEM Analysis

Material microscopic morphology characterization was obtained by using a scanning electron microscope (AKASHI SX-40, Akashi, Japan). BBR, QR, BQP, and BQS were, respectively, placed on the platinum tape, and then images were obtained under an excitation voltage of 20 kV under vacuum.

#### 4.2.6. Dissolution Experiment

The powder of QR (18 mg), BBR (20 mg), BQP (20 mg BBR + 18 mg QR) and BQS (38 mg) were respectively placed in 900 mL water and stirred at 50 rpm at 37 ± 0.5 °C through paddle-rotating method. A volume of 3 mL of the medium was withdrawn through a 0.45 μm membrane at time points of 15, 30, 60, 90, 120, 180, 240, 360 and 480 min and immediately added with 3 mL of fresh dissolution medium. There were 6 repeats under each point. The amount of dissolved BBR or QR was determined using the method described in Section 4.3.

### 4.3. Bioavailability Analysis

All animals were allowed to acclimatize for a week. Ninety healthy male C57BL/6N mice were stratified into eighteen groups (n = 5 per group) based on body weight to ensure a similar average weight across groups. Then, the animals fasted for 12 h. Mice in each group were intragastrically administered BQP or BQS (equivalent to 100 mg/kg BBR and 90 mg/kg QR) dissolved in water. Approximately 50 mg of liver tissue was obtained from each mouse at different time points (0.5, 1, 1.5, 2, 3, 6, 9, 12, and 24 h). Liver tissues collected were stored at −80 °C until analysis. The concentration of BBR and QR in the liver tissues was determined using the UHPLC–MS/MS method, as described below.

In this study, we established a mathematical model-assisted UHPLC-MS/MS method for the targeted quantification of BBR or QR in liver samples of normal C57BL/6N mice. The internal standard solution, containing Palmatine, was added to 50 mg liver tissue for homogenization. After centrifugation at 12,000 rpm for 10 min, 300 µL of the supernatant was transferred into 675 µL of methanol. After vortexing them for 30 s, the samples were centrifuged at 12,000 rpm for 10 min. The supernatant was taken as the final sample.

The LC-MS analysis was performed using a TripleTOF 4600 analyzer (AB SCIEX, Framingham, MA, USA). A C18 column was used for separation. The mobile phase comprised water (mobile phase A) and acetonitrile (mobile phase B). The gradient program was 0.00−0.50 min (10% B), 0.50−4.00 min (10−95% B), 4.00−6.00 min (95% B), 6.00−6.10 min (95–10% B), and 6.10−9.00 min (10% B). The experiment was performed in a negative mode of ionization. The quantitative ion pair of BBR, QR, and the internal standard (Palmatine) quantitative were *m*/*z* = 336.0/292.1, *m*/*z* = 303.1/229.1, and *m*/*z* = 352.1/308.1, respectively. The qualitative ion pair of BBR, QR, and the internal standard qualitative were *m*/*z* = 336.0/321.1, *m*/*z* = 303.1/153.1, and *m*/*z* = 352.1/322.1, respectively.

### 4.4. In Vitro Anti-Fibrosis Activity in LX-2 Cells

Lx-2 cells were cultured in DMEM at 37 °C in an atmosphere of 5% CO_2_. Once the cells reached 90–95% confluence, they were starved by incubating them in DMEM containing 2% FBS for 12 h. To create a liver fibrosis model, 2 ng/mL of TGF-β1 (R&D Systems, Minneapolis, MN, USA) was added to the cell culture. Additionally, the BQP and BQS (equivalent to a concentration of 10 μg/mL of BBR and 9.0 μg/mL of QR) were also added to the culture.

#### 4.4.1. Apoptosis

As described by Crowley et al. [55], the harvested cells were co-stained with fluorescein isothiocyanate (FITC) labeled Annexin V and propidium iodide (PI) (Beyotime Biotechnology, Shanghai, China) for 15 min at room temperature in the dark. Then, flow cytometric analysis was carried out on a flow cytometer (BD Biosciences, Franklin Lake, New Jersey, USA) and the results were analyzed using FlowJo_V10 software.

#### 4.4.2. Cell Cycle

The harvested cells were co-stained with propidium iodide (PI) (Beyotime Biotechnology, Shanghai, China) for 30 min at room temperature in the dark. The results were analyzed using FlowJo_V10 software.

#### 4.4.3. Immunofluorescence (IF) Analysis

The tyramide signal amplification (TSA) method was used for α-SMA (Affinity Biosciences, Liyang, Jiangsu, China) and collagen I (Affinity Biosciences, Liyang, Jiangsu, China) double staining. In brief, the samples were treated successively with rabbit primary antibodies and HRP-conjugated goat anti-rabbit secondary antibodies. A fluorescent reagent was incubated with the samples after washing to develop the color. The antibodies used are shown in Table S1.

### 4.5. In Vitro Analysis of HepG2 Cells

HepG2 (ATCC) cells were cultured in DMEM at 37 °C in an atmosphere of 5% CO_2_. The cells were seeded into six-well plants. We did not stimulate the cells until they reached 90–95% confluence. HepG2 cells were incubated with 0.5 mmol/L sodium oleate (SO, Sigma-Aldrich, St. Louis, MO, USA), together with the BQP and BQS (equivalent to 10 μg/mL of BBR and 9.0 μg/mL of QR). We harvested the cells after 12 h.

#### 4.5.1. Oil Red O Staining

After being fixed in paraformaldehyde, HepG2 cells were stained with 0.1% Oil Red O to detect lipid droplets. The results were analyzed using Fiji (ImageJ 1.53c) software.

#### 4.5.2. IF Analysis

The tyramide signal amplification (TSA) method was used for TNFα (Novus Biologicals, Littleton, CO, USA). IL-1β (ProteinTech Group, Chicago, IL, USA) and IL-6 (ProteinTech Group, Chicago, IL, USA) triple staining. The operation refers to the method mentioned in Section 4.4.3.

### 4.6. In Vivo Study

Six-week-old male C57BL/6N mice were purchased from the Vital River Laboratory Animal Technology (Beijing, China). All experimental procedures mentioned below were approved by the ethics committee of the Institute of Medicinal Biotechnology, Academy of Medical Sciences, and Peking Union Medical College (Beijing, China). The mice were allowed to acclimate in the specific-pathogen-free (SPF) grade animal facility (20 ± 1 °C on a 12 h light/dark cycle) for 7 days, with ad libitum access to water and food. The mice were randomly categorized into four groups (n = 10): a model group (MC, fed with Methionine and Choline-Deficient Diets, diet research, for six weeks) [56], a normal control group (NC, fed with a standard chow diet, for six weeks), a physical mixture group (BQP, fed with Methionine and Choline-Deficient Diets, diet research, for six weeks), and a salt group (BQS, fed with Methionine and Choline-Deficient Diets, diet research, for six weeks). Additionally, during the induction process, mice in the two drug administration groups were administered BQP (100 mg/kg/day of BBR and 90 mg/kg/day of QR) and BQS (190 mg/kg/day) by gavage, respectively. Both groups received a dosage equivalent to 100 mg/kg/day of BBR [57]; the mice in the MC and NC groups were administered a commensurable control vehicle intragastrically. The trial was conducted for two weeks.

At the end of the experiment, the mice were fasted for 12 h. Plasma was collected for biochemical analyses. The livers were harvested and weighed. We divided the liver into two parts; one was fixed with polyethylene, and the other was frozen at −80 °C.

#### 4.6.1. Biochemical Analysis

Plasma aspartate aminotransferase (AST), alanine aminotransferase (ALT), total bile acid (TBA), alkaline phosphatase (ALP), and gamma-glutamyltransferase (GGT) were measured using a TOSHIBA automatic biochemical analyzer (Toshiba, Tokyo, Japan), according to the manufacturer’s protocol and using commercially available kits (Biosino Biotechnology, Beijing, China).

#### 4.6.2. Tissue Staining

Liver samples were embedded in paraffin and then were sectioned into 4 μm thick sections. Hematoxylin–eosin, Sirius Red and Masson were used to stain the samples for microscopic observation.

#### 4.6.3. Immunohistochemistry (IHC) Analysis

The tyramide signal amplification (TSA) method was used for TNF-α, IL-1β double staining and for TGF-β, α-SMA, and collagen I triple staining. The operation refers to the method mentioned in Section 4.4.3.

#### 4.6.4. Enzyme-Linked Immunosorbent Assay

TG and CHO levels in mouse liver tissues were quantified using the TG assay kit and the CHO Assay Kit, respectively, both from Nanjing Jiancheng Bioengineering Institute (A110-1-1, A111-1-1, Nanjing, China). The liver tissue was accurately weighed and treated with the T-PER™ tissue protein extraction reagent at a ratio of 1:20 (g:mL). The samples were then centrifuged at 2500 rpm at 4 °C for 10 min. The supernatant obtained was used for subsequent analysis according to the manufacturer’s instructions.

### 4.7. ELISA Measurement

Levels of IL-1β and TNF-α from mouse liver tissue extracts or cultured supernatants of LX-2 cells were measured using ELISA kits for mouse or human IL-1β and TNF-α, respectively (MeilunBio, Dalian, China) according to the manufacturer’s protocols.

### 4.8. Quantitative Real-Time PCR (qPCR) Analysis

Total RNA was extracted from tissues or cells utilizing the RaPure Total RNA Kit (R4011, Magen, Guangzhou, China), strictly adhering to the manufacturer’s instructions. The quantitative polymerase chain reaction (qPCR) assays were conducted on the 7500 Fast Real-time PCR System (Thermo Fisher Scientific, Waltham, MA, USA) employing the HiScript^®^ II One Step gRT-PCR SYBR^®^ Green Kit (Q22101, Vazyme, Nanjing, China). The assays were performed under thermal cycling conditions as specified in the kit’s protocol. The sequences of primers used are listed in Table 1. Glyceraldehyde-3-phosphate dehydrogenase served as the internal reference gene. The qPCR data were analyzed using the comparative cycle threshold (Ct) method, and details regarding the qRT-PCR primers are provided in the accompanying table.

### 4.9. Network Pharmacology-Based Analysis

Initially, drug targets for BBR and QR were collected using keywords “berberine” and “quercetin” from databases like Drugbank, Swiss TargetPrediction, Targetnet, and batman-TCM. After removing duplicates, a substantial number of potential targets for both drugs were established. Targets related to hepatic fibrosis were then collected from the DisGenet and Gene Cards databases, utilizing criteria based on protein-coding relevance and scoring to refine the target list. Venn diagrams were employed to ascertain the intersection of drug targets with those associated with hepatic fibrosis, identifying crucial targets for further analysis. The intersecting genes were analyzed using the String database to construct a protein interaction network, which was then examined in Cytoscape to determine network dynamics. Core targets were identified using CentiScape, based on specific criteria for closeness, degree, and betweenness, and subsequently visualized. Enrichment analyses of these core targets were performed in the Metascape database for gene ontology (GO) and Kyoto Encyclopedia of Genes and Genomes (KEGG), elucidating their biological functions and pathways, with a focus on processes related to hepatic fibrosis.

### 4.10. Western Blot Analysis

Total liver proteins were extracted using RIPA lysis buffer, supplemented with protease and phosphatase inhibitors. Protein concentrations were determined using the BCA Protein Assay Kit (Thermo Fisher Scientific, Waltham, MA, USA). Twenty micrograms of total protein were subjected to electrophoresis on a 12% SDS-polyacrylamide gel and subsequently transferred to a polyvinylidene fluoride (PVDF) membrane. Following the transfer, the membrane was blocked with 5% non-fat milk and then incubated overnight at 4 °C with primary antibodies against phospho-Akt (1:1000, CST, Danvers, MA, USA), Akt (1:1000, Cell Signaling Technology Inc., Danvers, MA, USA), phospho-FoxO1 (1:1000, Cell Signaling Technology Inc., Danvers, MA, USA), Foxo-1 (1:1000, Cell Signaling Technology Inc., Danvers, MA, USA), Collagen I (1:3000, ProteinTech Group, Chicago, IL, USA), α-SMA (1:1000, Cell Signaling Technology Inc., Danvers, MA, USA) and β-actin (1:5000). After washing with TBST, the membrane was incubated with horseradish peroxidase-conjugated secondary antibodies (1:5000) at room temperature for one hour. The protein bands were visualized using a chemiluminescent detection reagent and an imaging system.

### 4.11. Statistical Analysis

All data are presented as mean ± standard error of mean with respect to the number of samples in each group. One-way ANOVA was used for comparisons among multiple groups, followed by Student’s *t*-test for independent group comparisons. Statistical significance was determined as follows: * *p* < 0.05; ** *p* < 0.01; *** *p* < 0.001; **** *p* < 0.0001; ^#^ *p* < 0.05; ^##^ *p* < 0.01; ^###^ *p* < 0.001. A *p*-value of <0.05 was considered statistically significant.

## 5. Conclusions

Liver fibrosis, a prevalent pathological condition associated with chronic liver diseases, currently lacks effective therapeutic options. Without timely and effective intervention, this condition may progress to several irreversible complications that significantly endanger human life. In this study, we successfully synthesized a novel compound, BBR–QR salt, which demonstrates outstanding biological and chemical properties. Comprehensive in vitro and in vivo studies have demonstrated that BQS possesses enhanced anti-inflammatory, anti-lipid accumulation, and anti-fibrotic capabilities compared to both the individual drugs and their physical mixture. This increased efficacy is likely mediated through complex molecular interactions and the modulation of critical biochemical pathways, particularly the Akt/FoxO1 pathway. Our results suggest that BQS interferes with the signaling cascade that promotes fibrogenesis, thus attenuating the pathological process at multiple levels. These findings underscore the potential of BQS as a potent therapeutic agent for liver fibrosis, highlighting its ability to act on key mechanistic pathways involved in the disease process. Given the promising results observed, further clinical trials are warranted to validate BQS’s efficacy and safety in human subjects. This could potentially lead to the development of a new, more effective treatment strategy for managing liver fibrosis, offering hope to millions affected by this debilitating condition.

## Figures and Tables

**Table 1:** Primer sequence information used for qRT–PCR.

Origin	Name	Forward (5′-3′)	Reverse (5′-3′)
Human	*IL-1β*	TATCATCTTTCAACACGCAGGACAG	TATCATCTTTCAACACGCAGGACAG
	*TNF-α*	GCCGTCTCCTACCAGACCAAG	ATGGGCTCATACCAGGGCTTG
	*IL-6*	ACAGACAGCCACTCACCTCTTC	AGTGCCTCTTTGCTGCTTTCAC
	*COL1A1*	AGGGCGACAGAGGCATAAAGG	AGGACCAGAGGCTCCAGAGG
	*α-SMA*	CCGGGAGAAAATGACTCAAA	GCAAGGCATAGCCCTCATAG
	*GAPDH*	GAACATCATCCCTGCCTCTACTGG	CCTCCGACGCCTGCTTCAC
Murine	*Tgfβ1*	CCGCTTCTGCTCCCACTCC	CATGTCGATGGTCTTGCAGGTG
	*Col1a1*	GGTCCTGCTGGTCCTGCTG	GAGAAGCCACGATGACCCTTTATG
	*Il-1β*	CAAACCTTTGACCTGGGCTGTC	GCCTGCCTGAAGCTCTTGTTG
	*Tnf-α*	GCCTCTTCTCATTCCTGCTTGTG	GTGTGAGGGTCTGGGCCATAG
	*α-SMA*	CTTCGTGACTACTGCCGAGC	AGGTGGTTTCGTGGATGCC
	*G* *apdh*	CTCCCACTCTTCCACCTTCG	TAGGGCCTCTCTTGCTCAGT

## Data Availability

The original contributions presented in the study are included in the article/Supplementary Materials.

## Supplementary Materials

The following supporting information can be downloaded at: https://www.mdpi.com/article/10.3390/ijms26052193/s1.
