# Supplementary material for: Enhancing the Therapeutic Efficacy of Berberine and Quercetin Through Salt Formulation for Liver Fibrosis Treatment
Source: Int J Mol Sci. 2025 Feb 28;26(5):2193. doi: 10.3390/ijms26052193 (PMC11899775; doi:10.3390/ijms26052193)
Supplement: Supplementary file 1 [file ijms-26-02193-s001.zip › Fig.S1.pdf]

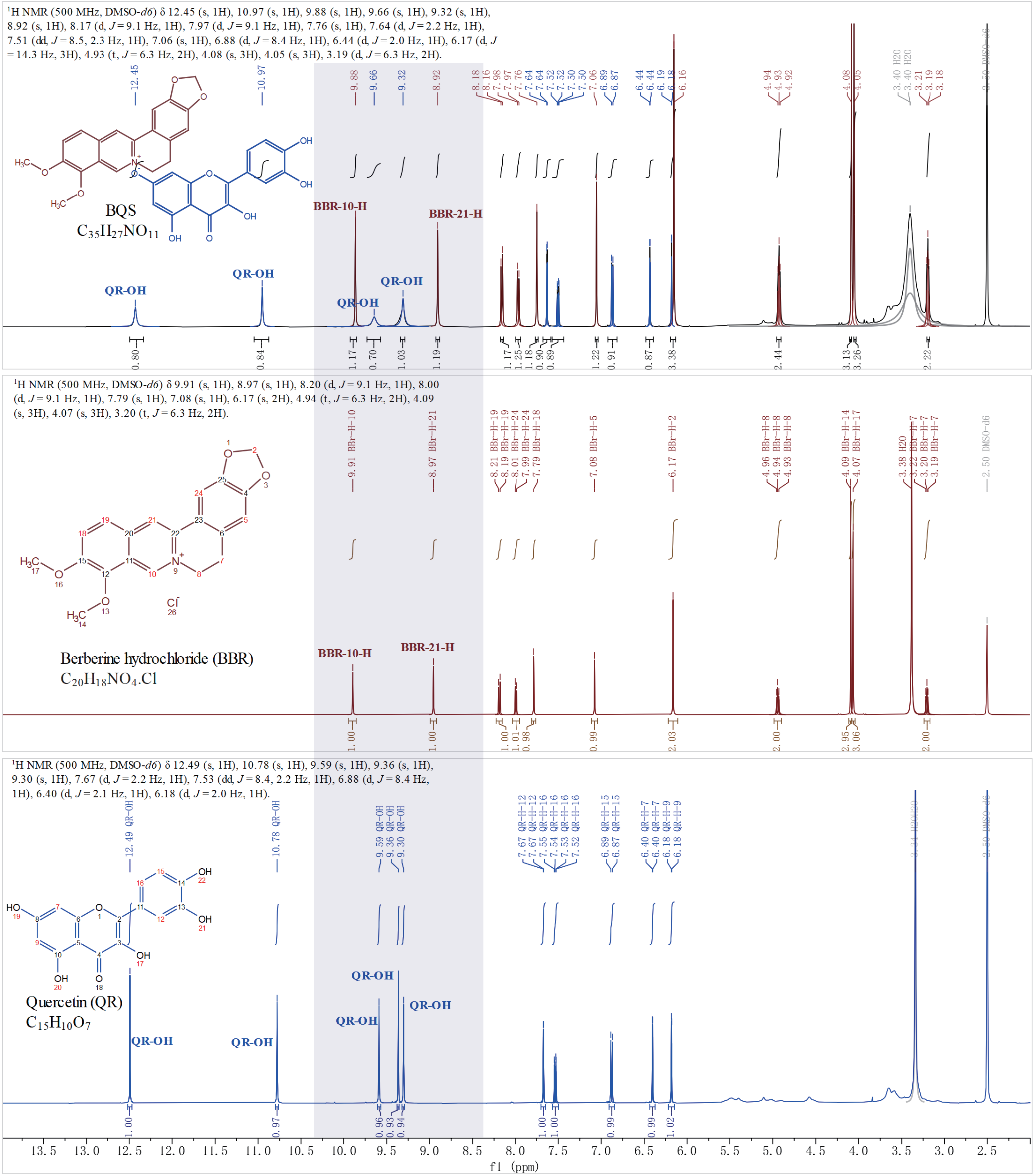
1H NMR (500 MHz, DMSO-d6) δ 12.45 (s, 1H), 10.97 (s, 1H), 9.88 (s, 1H), 9.66 (s, 1H), 9.32 (s, 1H), 8.92 (s, 1H), 8.17 (d, J = 9.1 Hz, 1H), 7.97 (d, J = 9.1 Hz, 1H), 7.76 (s, 1H), 7.64 (d, J = 2.2 Hz, 1H), 7.51 (dd, J = 8.5, 2.3 Hz, 1H), 7.06 (s, 1H), 6.88 (d, J = 8.4 Hz, 1H), 6.44 (d, J = 2.0 Hz, 1H), 6.17 (d, J = 14.3 Hz, 3H), 4.93 (t, J = 6.3 Hz, 2H), 4.08 (s, 3H), 4.05 (s, 3H), 3.19 (d, J = 6.3 Hz, 2H).
BQS
C35H27NO11
QR-OH
BBR-10-H
BBR-21-H
1H NMR (500 MHz, DMSO-d6) δ 9.91 (s, 1H), 8.97 (s, 1H), 8.20 (d, J = 9.1 Hz, 1H), 8.00 (d, J = 9.1 Hz, 1H), 7.79 (s, 1H), 7.08 (s, 1H), 6.17 (s, 2H), 4.94 (t, J = 6.3 Hz, 2H), 4.09 (s, 3H), 4.07 (s, 3H), 3.20 (t, J = 6.3 Hz, 2H).
Berberine hydrochloride (BBR)
C20H18NO4.Cl
1H NMR (500 MHz, DMSO-d6) δ 12.49 (s, 1H), 10.78 (s, 1H), 9.59 (s, 1H), 9.36 (s, 1H), 9.30 (s, 1H), 7.67 (d, J = 2.2 Hz, 1H), 7.53 (dd, J = 8.4, 2.2 Hz, 1H), 6.88 (d, J = 8.4 Hz, 1H), 6.40 (d, J = 2.1 Hz, 1H), 6.18 (d, J = 2.0 Hz, 1H).
Quercetin (QR)
C15H10O7
f1 (ppm)
