# Supplementary material for: Enhancing the Therapeutic Efficacy of Berberine and Quercetin Through Salt Formulation for Liver Fibrosis Treatment
Source: Int J Mol Sci. 2025 Feb 28;26(5):2193. doi: 10.3390/ijms26052193 (PMC11899775; doi:10.3390/ijms26052193)
Supplement: Supplementary file 1 [file ijms-26-02193-s001.zip › Table S1.pdf]

Table S1. Reagents used in immunofluorescence and WB.

	Name	Company	Product code
	α-SMA	Affinity	AF1032
	Collagen I	Affinity	AF7001
	TNF-α	Novus	NBP1-19532
	IL-1β	Proteintech	16806-1-AP
	IL-6	Proteintech	21865-1AP
	TGF-β	Proteintech	21898-1-AP
**Goat anti-rabbit IgG**	HRP-conjugated	Servicebio	GB23301
**Fluorescent dye**	Fitc-TSA	Servicebio	G1222
	CY3-TSA	Servicebio	G1223
	647-TSA	Servicebio	G1224
**Antibody for WB**	p-Akt	CST	9271
	Akt	CST	9272
	p-FoxO1	CST	9461
	FoxO1	CST	2880
	α-SMA	CST	19245
	Collagen Ⅰ	Proteintech	14695-1-AP
	β-actin	CST	4970
